# Graphene Oxide-Induced Substantial Strengthening of High-Entropy Alloy Revealed by Micropillar Compression and Molecular Dynamics Simulation

**DOI:** 10.34133/2022/9839403

**Published:** 2022-08-24

**Authors:** Wei Zhang, Hongcai Xie, Zhichao Ma, Hongwei Zhao, Luquan Ren

**Affiliations:** ^1^School of Mechanical and Aerospace Engineering, Jilin University, Changchun 130025, China; ^2^Key Laboratory of CNC Equipment Reliability Ministry of Education, Jilin University, Changchun 130025, China; ^3^Key Laboratory of Bionic Engineering Ministry of Education, Jilin University, Changchun 130025, China; ^4^Weihai Institute for Bionics, Jilin University, Weihai 264400, China

## Abstract

Plastic deformation mechanisms at micro/nanoscale of graphene oxide-reinforced high-entropy alloy composites (HEA/GO) remain unclear. In this study, small-scale mechanical behaviors were evaluated for HEA/GO composites with 0.0 wt.%, 0.3 wt.%, 0.6 wt.%, and 1.0 wt.% GO, consisting of compression testing on micropillar and molecular dynamics (MD) simulations on nanopillars. The experimental results uncovered that the composites exhibited a higher yield strength and flow stress compared with pure HEA micropillar, resulting from the GO reinforcement and grain refinement strengthening. This was also confirmed by the MD simulations of pure HEA and HEA/GO composite nanopillars. The immobile <100> interstitial dislocations also participated in the plastic deformation of composites, in contrast to pure HEA counterpart where only mobile 1/2 <111> perfect dislocations dominated deformation, leading to a higher yield strength for composite. Meanwhile, the MD simulations also revealed that the flow stress of composite nanopillar was significantly improved due to GO sheet effectively impeded dislocation movement. Furthermore, the mechanical properties of HEA/1.0 wt.% GO composite showed a slight reduction compared with HEA/0.6 wt.% GO composite. This correlated with the compositional segregation of Cr carbide and aggregation of GO sheets, indicative of lower work hardening rate in stress-strain curves of micropillar compression.

## 1. Introduction

Recently, high-entropy alloys (HEAs) have received considerable attention owing to the outstanding mechanical properties [1, 2]. Owing to the complex compositions, HEAs showed five core effects, containing high entropy effect in thermodynamics, lattice distortion effect in structure, slow diffusion effect in dynamics, cocktail effect in performance, and high stability in organization [3, 4]. According to the thermodynamic formula [5], the high mixing entropy enables Gibbs free energy to be low and thereby leads to a stable phase structure for HEAs. Great efforts have been made to enhance the mechanical properties of HEAs [6]. An effective method is to incorporate a reinforcing phase into the HEA matrix, obtaining HEA matrix composites [7]. The mechanical properties of the metal matrix composite are largely dependent on the volume fraction of the reinforcement. At present, the metal matrix composites have been successfully fabricated with the reinforcing phase of carbides, metal oxides, nitrides, and so on [8–10]. Graphene oxide (GO) and reduced graphene oxide (RGO), as derivatives of graphene, have been widely used as reinforcing phase in metal matrix [11], owing to the high strength, great toughness, excellent conductivity, and self-lubrication [12]. Liu et al. [13] investigated the graphene-reinforced Fe_50_Mn_30_Co_10_Cr_10_ HEA composites by spark plasma sintering (SPS) and mechanical alloying (MA). The results indicated that the composites showed a higher yield strength compared with HEA matrix. But the underlying deformation mechanisms of graphene oxide-reinforced HEA composites are still poorly understood, especially the plastic deformation mechanisms at the micro/nanoscale.

The plastic deformation mechanisms at the micro/nanoscale of HEA have been studied extensively in the last ten years by means of micropillar compression testing [14, 15]. The noteworthy point here is that the stress-strain responses obtained by the micropillar compression testing were proven significantly different from the macroscopic mechanical testing. As previously mentioned, micropillar compression testing, size effect, and stress burst were more obvious at a small scale [16, 17]. Combining the observation of the morphologies of deformed micropillars, researchers could quantitatively estimate the relationship between plastic deformation mechanisms and micromorphology. Molecular dynamics (MD) is also an effective method to acquire the deformation behaviors of nanopillars. For example, Zhang et al. [18] studied the plastic deformation mechanisms of HEA with body-centered cubic (BCC) phase based on the simulations of nanopillar compression. The results showed that the [111]-oriented HEA nanopillars exhibited a higher yield stress and flow stress compared with [100]- and [110]-oriented nanopillars. Moreover, Yaghoobi and Voyiadjis [19] found the size effect only depends on the size of nanopillars regardless of the value of the applied strain in the case of MD simulation of Al nanopillars. Thus, the results of MD simulation play a crucial role in revealing and forecasting the deformation mechanisms of GO-reinforced HEA matrix composites in this study. Micropillar compression testing combined with MD simulation of nanopillars serves as an excellent method to systematically study the deformation mechanisms of materials.

In this study, the bulk Fe_22_Co_24_Cr_20_Ni_23_Al_11_ HEA matrix composites with 0.0 wt.%, 0.3 wt.%, 0.6 wt.%, and 1.0 wt.% GO content were prepared by spark plasma sintering. To clearly reveal the mechanical properties of GO-reinforced HEA matrix composites at a small scale, the micropillar compression testing was conducted on the four samples. Meanwhile, MD simulations of nanopillars were employed to estimate the deformation mechanisms of composites. This study combined the micropillar compression testing with nanopillar MD simulations, with the objective of revealing the plastic deformation mechanisms of GO-reinforced HEA matrix composites at micro/nanoscale.

## 2. Results and Discussion

### 2.1. Microstructure of GO-Reinforced HEA Composites

**Figure 1:**
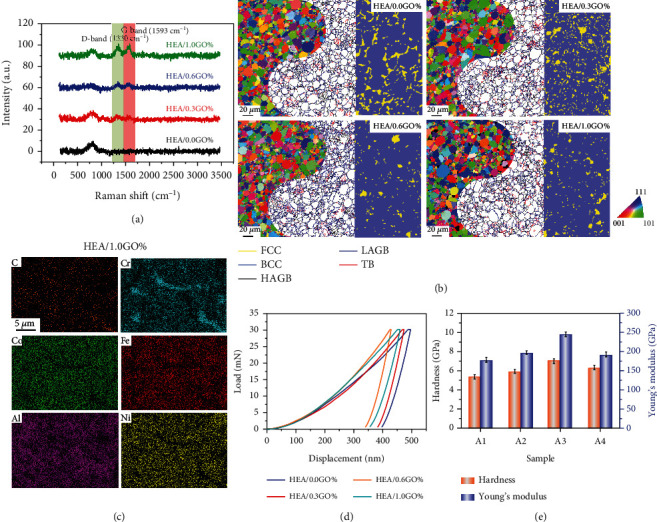
(a) Raman spectra of the HEA/0.0 wt.% GO, HEA/0.3 wt.% GO, HEA/0.6 wt.% GO, and HEA/1.0 wt.% GO composites (A1, A2, A3, and A4). (b) Corresponding EBSD orientation maps, grain boundary misorientation angle maps, and phase maps. (c) Elemental distribution of HEA/1.0 wt.% GO composite. (d) Load-displacement curves obtained by nanoindentation testing. (e) Hardness and Young's modulus of corresponding samples.

Figure 1(a) shows the Raman spectra of GO-reinforced HEA composites. HEA/0.0 wt.% GO, HEA/0.3 wt.% GO, HEA/0.6 wt.% GO, and HEA/1.0 wt.% GO composites were defined as A1, A2, A3, and A4, respectively. As compared with the HEA matrix, all the composites exhibited D-band and G-band. The Raman shifts of D-band and G-band were ~1330 cm^−1^ and ~1593 cm^−1^, respectively. This indicated that GO still existed in the composites after sintering. It is crucial for GO-reinforced composites to maintain the GO structure after sintering, owing to the structural integrity could be damaged in the sintering process. This significantly reduced the reinforced performance of GO. In practice, the degree of defects and disorder of carbon allotrope, such as graphite, graphene, and their oxide, can be detected by Raman spectrum. The D-band and G-band of these materials are usually found at ~1330 cm^−1^ and ~1580 cm^−1^ [20], respectively. The intensity of D-band increased with the degree of defects and disorder. Meanwhile, the relative intensity ratio of D-band to G-band (*I*_D_/*I*_G_) also increased. In Figure 1(a), the intensity of D-band and G-band increased with GO content. The *I*_D_/*I*_G_ values of A2, A3, and A4 were calculated to be 1.03, 1.05, and 1.05, respectively. It indicated that the crystallinity of GO was relatively high, though the fabrication process of composites brought a kind of destruction more or less to the GO sheets.

The EBSD results are shown in Figure 1(b), suggesting the distribution of grain orientation was random. Comparing the grain size of the A1, A2, A3, and A4, one can see that the grain size decreased significantly with the incorporation of GO reinforcement. In particular, the grain size of composites undergoes a considerable reduction with the increase of GO content. It could be concluded that the incorporation of GO reinforcement could refine the grains of the HEA matrix, which was caused by the pinning effect of the uniformly distributed GO sheets in composites [21]. Thus, the grain growth was effectively inhibited by the dispersed GO sheets. However, the grain sizes of A3 and A4 were similar. Combined with the black regions in EBSD orientation maps (A4), it could be determined that the GO sheets agglomerated in the sintering process. This phenomenon significantly reduced the strengthening effect of GO on HEA matrix and pinning effect of GO on grain boundary, leading to the grain growth of A4 composites which was not effectively restricted. Moreover, a large number of nanoscale particles existed in the vicinity of large-angle grain boundaries, which were primarily surrounded by the low-angle grain boundaries. As mentioned earlier, the uniformly dispersed GO sheets restricted the grain growth of nanoscale particles. On the other hand, the grains without enough time to grow up in the sintering process due to the prototyping and sintering of materials were rapid by using the SPS [22]. As previously reported, the bulk composites prepared by SPS could acquire ultrafine grains or even nanocrystalline via effectively controlling the recrystallization and grain growth [23]. It was also found that all the samples regardless of the content of GO show large-angle grain boundaries (HAGBs), low-angle grain boundaries (LAGBs), and twin grain boundaries (TBs). Large-sized and small-sized grains were mainly surrounded by the HAGBs and LAGBs, respectively. It is evident that all the samples exhibited a dual-phase structure with face-centered cubic (FCC) and body-centered cubic (BCC) phases. The proportion of BCC phase is much higher than that of FCC phase, and the volume fraction of FCC phase was further reduced with the incorporation of GO in HEA matrix. Unlike the rod-shaped FCC phase in the HEA matrix, the FCC phase in the composites was observed with a smaller nanostructure. This phenomenon is consistent with the EBSD grain boundaries maps, in which considerable nanoscale grains were found.

For the A4, the voids formed in the sintering process due to the agglomeration of GO sheets, which obviously reduced the strengthening effect of GO reinforcement. Apart from this factor, compositional segregation is another reason for the existence of voids. As shown in Figure 1(c), the Cr and C elements were rich in the vicinity of the grain boundaries, which indicates that the Cr carbide was formed in the A4. It is well known that the electronegativity difference of Cr and C is large, leading to the intermetallic compound which is easy to form. Besides, the Cr element has large negative mixing enthalpy with C element and hence are potential candidates for the synthesis of intermetallic compound. In fact, carbide of Cr was always observed in the graphene/metal composites simultaneously including Cr and C elements [24]. The precipitation of Cr carbide at the grain boundaries influences the mechanical properties of materials [25].

### 2.2. Nanoindentation Testing of Bulk GO-Reinforced HEA Composites

Prior to the micropillar compression testing, nanoindentation testing was conducted to have a preliminary understanding of the mechanical properties of the bulk samples. Figure 1(d) shows the typical load-depth (*P*‐*h*) curves, and the values of hardness and Young's modulus are shown in Figure 1(e). The hardness of A1, A2, A3, and A4 were calculated to be 5.39, 5.92, 7.09, and 6.31 GPa, respectively. It was revealed that the hardness values exhibited a rising tendency with the GO content increased. The hardness reached a maximum value in the case of A3. The reason why the composites showed higher hardness than HEA matrix was that the addition of GO provided a grain refinement strengthening effect to HEA matrix. Grain refinement strengthening mechanism plays an important role in metal matrix composites [26]. The number of grain boundaries in the material increased with the grain size decreased, leading to more energy which was required for crack propagation. Then, the crack propagation needs to pass through more grain boundaries, which made it difficult for the material to deform. Meanwhile, the number of grains on the unit cross section was large in the sample with small-sized grains, resulting in a greater deformation resistance. Besides, the hardness of A4 was slightly decreased as compared with A3 due to the excess incorporation of GO reinforcement to HEA matrix. GO nanosheets are prone to agglomerate, which seriously weakens the pinning effect on the grain boundary. In particular, the agglomeration of GO reduced the interfacial bonding force of GO reinforcement and HEA matrix, causing some voids formed in the materials. This was also confirmed by the EBSD orientation map in Figure 1(b). Moreover, the Young's modulus of the analyzed samples shows a similar tendency with the hardness.

### 2.3. Micropillar Compression of GO-Reinforced HEA Composites

**Figure 2:**
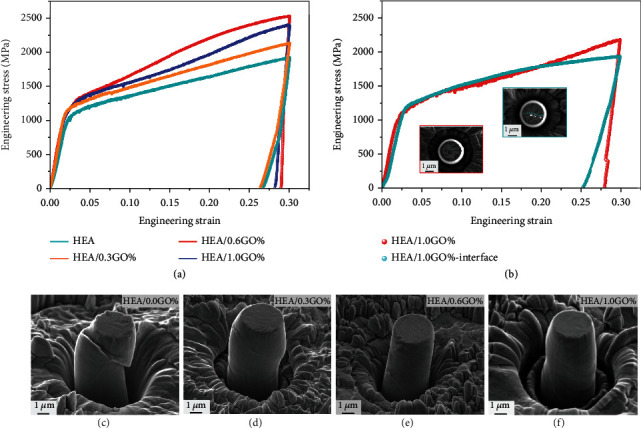
(a) Compressive stress-strain curves of the HEA/0.0 wt.% GO, HEA/0.3 wt.% GO, HEA/0.6 wt.% GO, and HEA/1.0 wt.% GO composite micropillars. (b) Compressive stress-strain curves of the HEA/1.0 wt.% GO composite without interface and with interface, the insets were top morphologies of micropillars. (c–f) SEM images of the corresponding deformed micropillars.

To obtain a direct and quantitative measurement of the micromechanical properties at specific site, the uniaxial micropillar compression testing was conducted on the A1, A2, A3, and A4. As previously reported, the grain boundary and crystal orientation of micropillars were two factors that influenced the mechanical properties of materials [27]. In this study, the micropillars were fabricated in a single crystal to eliminate the effect of grain boundaries. All the micropillars were prepared with the same [001] orientation, which also avoided the variation of mechanical properties induced by the different orientations.  Figure 2(a) shows the representative stress-strain curves of HEA/GO composites obtained by micropillar compression testing, in which the micropillars were not compressed to fracture and the strain was 0.30. It is obvious that the HEA/GO composite micropillars exhibited a higher yield strength and flow stress compared with HEA matrix. The measured and calculated values are listed in Table 1. The strengthening capabilities of the GO were improved with the content increased when the content of GO is <0.6 wt.%. However, the strengthening efficiency of A4 was much lower than A3, and this result was consistent with the nanoindentation testing. As mentioned above, the mechanical properties of A4 were weakened due to the aggregation of GO nanosheets and compositional segregation at the grain boundary. But the essential cause of it was still unrevealed. The strain hardening rates of the four samples were calculated from the stress-strain curves, and the trend was consistent with the flow stress and strengthening efficiency. In particular, the strengthening efficiency of A3 was much higher than A4, though the yield strength was similar. Based on the observations above, the A4 exhibited lower strengthening efficiency was attributed to the low strain hardening rate. In order to further confirm this analysis, micropillar compression testing was also conducted on the A4 without interface and with interface. The interface was referred to the region-enriched Cr carbon. As can be seen from Figure 2(b), the yield strength and flow stress of the two samples were approximately same when the strain was lower than 0.20. However, the micropillar containing interface exhibited lower flow stress compared with the micropillar without interface when the strain was larger than 0.2, which was attributed to the obvious difference of strain hardening. Variation in strain hardening rate profoundly affected the mechanical properties and deformation mechanisms of materials [28]. In the micropillar compression testing of HEA samples, stress burst has been frequently found in the stress-strain curves, which represented the dislocation annihilated in the plastic deformation [29]. Note that such large bursts were not observed in the curves of Figure 2, and the four curves in this study exhibited weak stress burst. This is probably because the HEA sample used in this paper has substantial BCC structure and rare FCC structure. The plastic deformation of HEA with BCC structure is weaker compared with FCC structure [30]; thus, the dislocation movement is less active.

Figures 2(c)–2(f) show typical postcompression SEM images of A1, A2, A3, and A4 micropillars. All the compressed micropillars showed clear crystallographic slip lines on the surface of the pillars regardless of GO content. The deformed pure HEA micropillar showed only a single slip trace close to the upper end of the micropillars and no evidence of multiple slip behaviors, indicative of localized deformation. This was attributed to the little strain hardening rate acquired from the corresponding stress-strain curves [31]. In contrast to the pure HEA micropillar, composite micropillars undergone a transition from localized plasticity to homogeneous deformation. A2 micropillar exhibited a combination of localized and homogeneous deformation, while A3 micropillar only showed homogeneous deformation. Because the avalanche occurs on A3 micropillar, more slip systems need to be activated [27]. In addition, the A4 micropillars studied here showed necking, as shown in Figure 2(f).

### 2.4. Molecular Dynamics Simulation of GO-Reinforced HEA Composite Nanopillars

**Figure 3:**
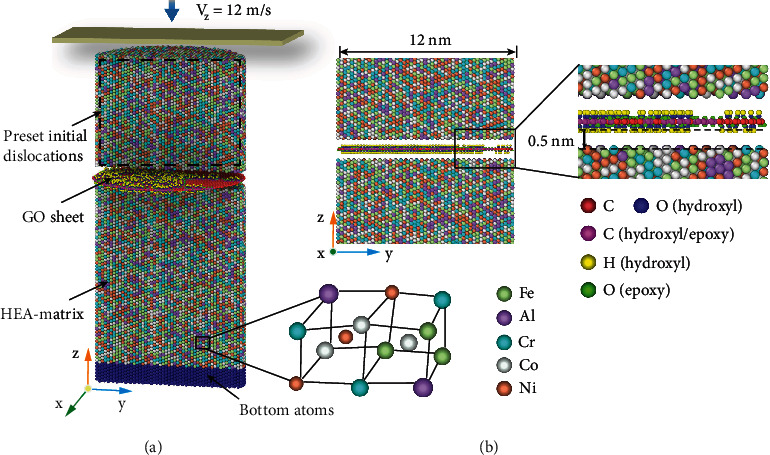
(a) Atomic configurations of HEA/GO nanopillar. (b) Local atomic structure of HEA matrix and GO sheet.

In order to further capture the underlying strengthening mechanisms observed in the micropillar compression testing, MD simulations of uniaxial compression were conducted on the HEA/GO composite nanopillars by means of LAMMPS software [32]. The OVITO software was applied to visualize the MD results during the nanopillar compression [33]. The atomic configurations of the HEA/GO nanopillar were constructed, which involved a diameter of 12 nm and an aspect ratio of 2.0, as illustrated in Figure 3(a). The [-110], [-1-12], and [111] crystallographic directions were configured along *x*, *y*, and *z* axes, respectively.

**Figure 4:**
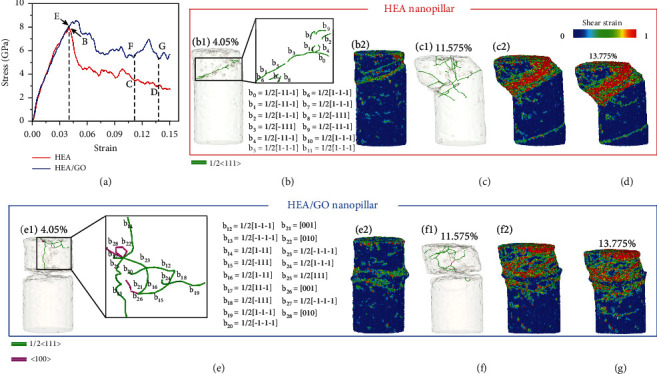
Strengthening mechanisms of HEA/GO nanopillar captured from MD simulations: (a) stress-strain curves; nucleated dislocations and atomic shear strain of HEA nanopillar at strain 4.05% (b1 and b2) and 11.575% (c1 and c2); (d) atomic shear strain of HEA nanopillar at strain 13.775%; nucleated dislocations and atomic shear strain of HEA/GO nanopillar at strain 4.05% (e1 and e2) and 11.575% (f1 and f2); (g) atomic shear strain of HEA/GO nanopillar at strain 13.775%. All HEA atoms in (b2, c2, d, e2, f2, and g) were colored based on the atomic shear strain, respectively. Nucleated dislocations in nanopillars were captured by DXA.

Figure 4(a) presents the stress-strain curves of pure HEA and HEA/GO nanopillar. The compressive procedure undergoes a transition from the elastic regime to the plastic yield regime. Compared with the curve of HEA/GO nanopillar, the pure HEA nanopillar first reached yield strength at strain 4.05% (corresponding to point B), indicating that the dislocation source was fully activated earlier. Obviously, HEA/GO composite nanopillar exhibited a higher yield stress and flow stress compared with pure HEA nanopillar. Unlike the tracks of slipping plane on pure HEA nanopillar, no pronounced dislocation slip trajectory was observed in HEA/GO composite nanopillar, as shown in Figures 4(b2) and 4(e2). To figure out the reason of this phenomenon, DXA was utilized to identify the dislocation structures of the two nanopillars at strain 4.05% in the curves, as shown in Figures 4(b1) and 4(e1). It is obvious that only 1/2 <111> perfect dislocations were observed in pure HEA nanopillar. Such perfect dislocations were mobile. Comparatively, the mobile 1/2 <111> perfect dislocations as well as <100> interstitial dislocations were found in HEA/GO composite nanopillar, as evidenced by the local enlarged drawing of Figure 4(e1). The latter can be formed by attracting and reacting of two 1/2 <111> perfect dislocations on the {110} plane. In the local enlarged drawing of Figure 4(e1), a typical dislocation reaction [34] during the compression of HEA/GO nanopillar was observed.

(1) $$\begin{array}{c} \left\{b_{15}+b_{16}⟶b_{26},\frac{1}{2}\left[-111\right]+\frac{1}{2}\left[1-11\right]⟶\left[001\right]\right\}. \end{array}$$

The <001> interstitial dislocation was formed by this dislocation reaction according to Equation (1), which exhibited a remarkable structural stability and resistance to dissociation. On the one hand, this stability stemmed from the reduction of elastic energy due to the dislocation reaction. On the other hand, it can be ascribed to the fact that the interstitial dislocation is located on a nonslipping {001} plane. Consequently, the immobile <100> interstitial dislocations play a crucial role in effectively pinning dislocation motion [35]. Besides, the two 1/2 <111> perfect dislocations involved in the reaction were anchored by the immobile <100> interstitial dislocation. The vast majority of 1/2 <111> dislocations motion in HEA/GO nanopillar was directly or indirectly restricted by <100> interstitial dislocations. Only after reaching a higher stress level can the immobile <100> dislocations reversely react and decompose to form mobile 1/2 <111> dislocations, thereby completely activating the dislocation source. This delivered a clear verdict as to why HEA/GO nanopillar exhibited a higher yield strength.

In the plastic flow regime, HEA/GO nanopillar rendered a higher flow stress level than that of pure HEA nanopillars. At strain 11. 575%, a slip band induced by massive dislocations on the same slip plane was observed in pure HEA nanopillar, as shown in Figure 4(c). A similar behavior was also observed by Zhang et al. and Huang et al. [18, 36]. Meanwhile, the dislocations gradually extended to the lower half of the nanopillar. Comparatively, the dislocations in HEA/GO nanopillar were mainly distributed in the upper side, and rare 1/2 <111> dislocations were seen running through the GO sheet to the lower half of the nanopillar, as shown in Figure 4(f), suggesting that the plastic deformation primarily occurred in the upper region of HEA/GO nanopillar. This observation indicated that GO substantially affected the deformation mechanisms of nanopillars and considerably limited the movement of dislocations. An analogous blocking effect on dislocation propagation was also detected in copper nanopillars [37]. At strain 13. 775%, as shown in Figure 4(g), a dislocation slip can be captured in the HEA matrix below the GO sheet, indicating that the whole nanopillar undergone plastic deformation. In addition, the pure HEA nanopillar exhibited more intense plastic deformation as compared with HEA/GO nanopillar regardless of point C and point D. Thus, the higher flow stress of HEA/GO nanopillar can be attributed to the dislocation congestion above the GO sheet induced by the strong HEA/GO interface effect.

## 3. Conclusions

In the present study, plastic deformation mechanisms at micro/nanoscale of graphene oxide-reinforced high-entropy alloy composites were studied by means of micropillar compression testing and MD simulation of nanopillars. To study the influence of GO on the mechanical properties of HEA matrix, the conclusions can be summarized as follows:

The uniaxial micropillar compression testing was conducted on the HEA/0.0 wt.% GO, HEA/0.3 wt.% GO, HEA/0.6 wt.% GO, and HEA/1.0 wt.% GO composites (A1, A2, A3, and A4). The stress-strain response revealed that the composites exhibited a higher yield strength and flow stress compared with pure HEA matrix, resulting from the grain refinement strengthening and inhibition effect of GO to dislocation movement. The yield strength of composites climbed up and then declines with increasing GO, which was the largest when the volume fraction of GO was 0.6 wt.%. It is worth noting that the mechanical properties of A4 showed a slight reduction compared with A3. This correlated with that of the aggregation of GO sheets and compositional segregation of Cr carbide in the vicinity of grain boundaries. Furthermore, the compression testing was also conducted on the micropillar with and without grain boundary, respectively. It is found that the micropillar containing grain boundary (with Cr carbide) exhibited lower strain hardening.

The MD simulations were performed on the pure HEA and HEA/GO composite nanopillars. The HEA/GO composite exhibited a higher yield strength and flow stress compared with pure HEA nanopillar, which was consistent with the experimental results of micropillar compression. Only mobile 1/2 <111> dislocations were observed in pure HEA nanopillar, while the mobile 1/2 <111> dislocations as well as immobile <100> interstitial dislocations were found in HEA/GO composite nanopillar. In contrast to the pure HEA nanopillar, the dislocations generally are incapable of running through the GO sheet; thus, the dislocations in HEA/GO nanopillar were mainly distributed in the upper side. This observation indicated that the GO improved the mechanical properties of composite through limiting the movement of dislocations.

## 4. Methods

### 4.1. Sample Preparation

The high-entropy alloy (HEA) Fe_22_Co_24_Cr_20_Ni_23_Al_11_ (atomic percent, at.%) powers were used to synthesize metal matrix. The average diameter of HEA powder is about 25 *μ*m. Graphene oxide shows 0.2-10 *μ*m in length and 1 *μ*m in thickness. The GO sheets and Fe_22_Co_24_Cr_20_Ni_23_Al_11_ powders were mixed in distilled water for 1 h and dispersed by ultrasonic for 2 h. The ultrasonic vibrates with a frequency of 30 kHz. To acquire a mixed suspension, the dispersed HEA and GO suspension were added in a beaker together and kept mechanically stirred for 1 h. Then, the hybrid suspension was filtered in a filter. The mixtures were dried thoroughly in a vacuum oven at 50°C. Mixture powers of HEA with a volume fraction of 0.0 wt.%, 0.3 wt.%, 0.6 wt.%, and 1.0 wt.% GO were prepared. The bulk samples were synthesized by spark plasma sintering (LABOX-1575). The samples were sintered at 900°C for 5 min. The pressure was 45 MPa, and the vacuum was 10 Pa. The heating rate was controlled to be about 100°C/min. The prepared bulk samples show dimensions of *Ø*30 × 10 mm.

### 4.2. Characterization

Raman spectroscopy (DXR3) with laser wavelength of 532 nm was utilized to characterize the GO in bulk composites. To obtain the structural information, the electron backscatter diffraction (EBSD) observation was performed in a field emission scanning electron microscopy (SEM, Zeiss-Crossbeam XB 1540). Before the fabrication of micropillar, the EBSD also was used to locate the site of micropillars. The EBSD information was analyzed by MTEX and MATLAB software. The morphology of the micropillars before and after compression was captured by SEM. Surface hardness of the bulk samples were measured in a nanoindenter (Anton Paar) equipped with a diamond Berkovich tip. The indentation tests were performed in a load-controlled mode, and the maximum load was 30 mN. The loading rate of tip was 1.5 mN/s. Five indentations were measured for each sample. The elemental distribution was obtained by the energy-dispersive X-ray spectroscopy (EDS) equipped on SEM.

### 4.3. Micropillar Compression Tests

The bulk composites were Ar^+^ ion polished prior to the EBSD analysis. The micropillars were prepared on a polished EBSD sample using a dual beam-focused ion beam (FIB, FEI Helios Nanolab 600i). All pillars were fabricated in single crystal with [001] orientation to eliminate the effects of grain boundary and orientation. Three micropillars were prepared for each composite. The cutting process was divided into two steps. First, currents ranged from 5 nA to 600 pA were selected for a coarse milling, aiming to remove material quickly. Then, a small current of 10 pA was used to mill desired shape of micropillars. Unavoidably, the prepared micropillar with a taper ranged from 2° to 4° due to the damage of Ga^+^ ion. The micropillars with a diameter of 3 *μ*m and a height-diameter ratio of 2 were milled for subsequent compression testing. The diameter of pit is 25 *μ*m, which provides a sufficient clearance for flat indenter (diameter is 10 *μ*m) compression. The micropillar compression testing was conducted on a nanoindenter (Hysitron PI 85L) with a flat punch inside the chamber of SEM. A displacement control mode was used in the testing, and the strain rate was 2 × 10^−3^ s^−1^. The maximum engineering strain was 0.3. The height and top diameters of original micropillars are chosen to calculate the values of engineering stress and strain, which agrees well with previous reports [38].

### 4.4. Simulation Details and Methodology

The GO model was divided into two steps to construct. First, the initial data file was referred to the method in the previous report [39], and the method was proposed on the basis of the local reactivity of graphene systems. Then, it was modified and edited in the Moltemplate software [40]. The C : O ratios were set to 3.0. Obviously, the epoxy and hydroxyl groups can be observed on the upper and lower surfaces of GO sheet, as shown in Figure 4(b). The HEA matrix atoms within 0.5 nm from the GO atoms were removed. Periodic boundary conditions were applied in the *z*-direction, and free boundary conditions were used in *x*- and *y*-directions. The time step was 1 fs. The simulation procedure can be categorized into equilibrated and compressive loading stages. Prior to the compressive loading, the initial model was optimized be means of the conjugate gradient (CG) technique [41]. In most micro/nano pillars, there are profuse dislocations before compressive loading. To make the simulation closer to the experimental conditions, initial dislocations were introduced into the nanopillars by creating vacancies and subsequent high-temperature heat treatment [42]. The 10% vacancy atoms in the upper end of the nanopillars were randomly deleted to capture the interaction between dislocations and HEA/GO interface, which were marked by a black dashed box in Figure 4(a). Then, the isothermal-isobaric (NPT) ensemble was applied to control the temperature and pressure of the nanopillars in high-temperature annealing and quenching (heating from 300 K to 1000 K for a duration of 50 ps, annealing at 1000 K for 50 ps and quenching to 300 K for 50 ps). Besides, the system was additionally annealed at a pressure of 0 bar and a temperature of 300 K to obtain an equilibrium system. Then, the nanopillars were compressed along the *z*-axis by a virtual rigid plane at a rate of 12 m/s under the canonical (NVT) ensemble. Notably, such a virtual plane was realized by the repulsive force model, as shown in Equation (2), which has been extensively employed in previous studies [19, 35, 43]. For comparison, the pure HEA nanopillar was compressed under the same simulation conditions.

(2) $$\begin{array}{c} F\left(r\right)=\left\{\begin{array}{cc} -K{\left(r-r_{c}\right)}^{2}, & r<r_{c},\\ 0, & r\geq r_{c}, \end{array}\right. \end{array}$$

where *K* is the specified force constant, which *r* is selected as 1 eV/Å^2^. *r* is the distance from the atom to the virtual plane, which is chosen as 3 Å. *r*_*c*_ is the cutoff distance.

The interatomic interaction within the Al-Co-Cr-Fe-Ni system was expressed by the embedded atom method (EAM) potential from the EAM database developed by Zhou et al. [44], which has been widely used in previous reports regarding HEA system [18, 45–47]. The interatomic interaction inside GO sheet was described by the OPLS-AA force field [48, 49]. The long-range interactions were captured by the particle-particle particle-mesh (PPPM) method. In addition, the Lorentz-Berthelot mixing rule was employed to describe the Lennard-Jones (L-J) atomic interactions inside HEA matrix and GO sheet [50, 51]. The dislocation extraction algorithm (DXA) technique [52] was employed to diagnose dislocation movements in nanopillars. The atomic von Mises shear strain was used to track the slip traces of atoms in nanopillars [53]. The stress along the loading direction can be obtained by adding the von Mises stresses of the local atoms and dividing by the volume of the deformed nanopillars [42, 54, 55].

## Figures and Tables

**Table 1:** Mechanical properties of the HEA/GO composites obtained by micropillar compression testing.

Sample	Yield strength (MPa)	Flow stress		Strain hardening rate (MPa)	Strengthening efficiency
		Strain at 0.05	Strain at 0.30		
HEA/0.0 wt.% GO	1064 ± 53	1191 ± 26	1898 ± 27	2828	/
HEA/0.3 wt.% GO	1148 ± 27	1297 ± 44	2108 ± 21	3244	14.71%
HEA/0.6 wt.% GO	1305 ± 31	1389 ± 35	2496 ± 46	4428	56.57%
HEA/1.0 wt.% GO	1209 ± 35	1366 ± 42	2354 ± 39	3952	39.75%

## Data Availability

The data that support the findings of this study are available from the corresponding author on reasonable request.
